# The Translation and Adaptation Of The Child Behaviour Checklist (CBCL) To Azerbaijan Culture

**DOI:** 10.1192/j.eurpsy.2023.1544

**Published:** 2023-07-19

**Authors:** N. Osmanli, T. Ergene, A. Ozer

**Affiliations:** 1 World Health Organization CO, BAKU, Azerbaijan; 2 Hacettepe University, Ankara, Türkiye

## Abstract

**Introduction:**

The lack of valid and reliable screening tools is the one of the significant barriers to the extension of studies in child and adolescent mental health in Azerbaijan culture.

**Objectives:**

The aim of this study is to adapt the Child Behavior Checklist (CBCL 6-18) to Azerbaijan culture.

**Methods:**

Study group of the research is consist of 1232 (630 female, 599 male) children and adolescents between the ages of 6-17 studying in classes 1 to 11 in Azerbaijan.The ability to explain the data obtained from Azerbaijani Version of CBCL by theoretical model was examined by confirmatory factor analysis (CFA).

**Results:**

The RMSEA index was calculated as .09 for the one-dimensional alternative model and Comparative Fit Index as .93 for CBCL Azerbaijani Version. It was concluded that the alternative one-dimensional model, where a series of indexes were evaluated together, has an acceptable fit.Internal consistency coefficients were calculated as .94 for Total Problems, .87 for Internalizationand .87 for Externatilazation. The internal consistency coefficients for the empirically based problem subtests varied between .62 and .86.Correlations with total score of total syndrome subtests of the checklist were calculated for female and male students and 6-11/12-17 ages, and a positive and significant correlation was found for female and male students and 6-11/12-17 age groups (p <.05). The correlations between the Total Problem and all syndrome subtests ranged from .68 to .88 for boys and .67 to .88 for girls. Furthermore, a strong correlation (r> .70) between Anxiety/Depression, Social Withdrawn/Depression and Internalizing Problems and between Aggressive Behavior, Delinquent Behaviour problem subtests and Externalizing Problems, was detected.It was found that Externalizing Problems and Aggressive Behavior subtests of boys has a significantly higher average than girls (p<.05).It was concluded that the scores of Social Withdrawn/Depression and Internalizing Problems of girls and Social Problems and Thought Problems of boys increases, and Social Withdrawn/Depression and Internalizing Problems scores of boys decreases as the age increases.

**Conclusions:**

Considering that a row of indexes is evaluated together in examining model fit, it can be said that the model consisting of eight factors has an acceptable fit.

**Disclosure of Interest:**

None Declared

